# CROF-Net: A Robust Algorithm for Simulation-Measurement Cross-Domain Few-Shot HRRP Target Recognition

**DOI:** 10.3390/s26175404

**Published:** 2026-08-26

**Authors:** Xiaoyu Xu, Mingbo Zhu, Keyuan Yu, Kangsheng Liu

**Affiliations:** 1 Naval Aviation University, Yantai 264001, China; 17353396821@163.com (X.X.); mb_zhu@126.com (M.Z.); 17861169806@163.com (K.L.); 2 Key Laboratory of Sea-Air Information Perception and Processing Technology of Shandong Provincial, Yantai 264001, China; 3 National Key Laboratory of Millimeter-Wave and Terahertz Remote Sensing, Yantai 264001, China

**Keywords:** high-resolution range profile, radar automatic target recognition, cross-domain few-shot learning, domain adaptation, orthogonal sub-center metric learning

## Abstract

**Highlights:**

**What are the main findings?**

- An improved multi-sub-center ArcFace metric originally designed for computer vision tasks is tailored for 1D radar HRRP sequences, equipped with orthogonal regularization to mitigate intra-class scattering aliasing and simulation-measurement cross-domain negative transfer under few-shot conditions.
- A scattering-peak-aware 1D-Conformer backbone with instance normalization is developed, combined with low-order empirical moment matching for simulation-measurement domain alignment. Evaluated on the public SAMPLE and MTDSP datasets, it achieves superior recognition performance and robustness against electromagnetic distortions compared to state-of-the-art models.

**What are the implications of the main findings?**

- This study extends hyperspherical angular metric learning from 2D vision images to 1D radar time-series signals, providing a feasible cross-field migration paradigm for remote sensing signal processing.
- The proposed cross-domain few-shot learning framework effectively addresses the scarcity of measured data for non-cooperative targets, offering a robust solution for radar automatic target recognition under complex electromagnetic environments.

**Abstract:**

Aiming at limited measured HRRP samples of non-cooperative targets and severe distribution discrepancy between simulated and real echoes, this paper proposes CROF-Net, a robust cross-domain few-shot recognition framework for complex electromagnetic environments. It first corrects simulated data distribution via low-order moment matching and enhances few-shot measured samples for domain consistency. A 1D-Conformer backbone embedded with instance normalization and scattering-peak-aware modules extracts robust local and global features from distorted HRRP signals. A reliability-aware prototype calibration module achieves fine-grained class-level alignment. To handle attitude-induced intra-class multimodal distribution, we design an orthogonal-constrained sub-center ArcFace metric space to broaden classification boundaries and relieve negative transfer and catastrophic forgetting. Evaluated on SAMPLE and MTDSP datasets, CROF-Net achieves higher accuracy and stronger anti-interference capability than existing methods, presenting great research value and engineering application prospects.

## 1. Introduction

Radar automatic target recognition (RATR) is a core supporting technology for air defense early warning and precision guidance [1]. Compared to two-dimensional synthetic aperture radar (SAR) images, radar high-resolution range profile (HRRP) features easy data acquisition, low processing cost, and fast response speed [2]. As a mainstream data form for non-cooperative target recognition, HRRP can accurately characterize the spatial distribution and structural features of strong electromagnetic scattering centers of targets along the radar line-of-sight (LoS) direction [3].

Aiming at three practical bottlenecks in radar HRRP recognition, including scarce measured samples, simulation-measurement domain distribution shift, and complex electromagnetic distortion interference, this paper proposes a cross-domain robust orthogonal few-shot network (CROF-Net). A progressive complete algorithm framework is constructed following the pipeline of “data-level domain adaptation—robust temporal feature modeling—prototype distribution correction—metric classification optimization." The core innovations are summarized as follows:

(1). Physically guided alignment and disturbance augmentation
  Low-order moment matching is adopted to globally calibrate HRRP echo waveforms and narrow the distribution gap between simulated and measured data. Meanwhile, occlusion and jamming-based augmentation strategies are applied to expand limited measured samples, reducing the model’s reliance on high-SNR data and establishing robust pre-training priors for subsequent feature learning and cross-domain adaptation. Different from mainstream complex adversarial alignment and single statistical matching schemes, this lightweight moment alignment strategy introduces no extra computational overhead and effectively suppresses negative transfer under extremely limited measured samples.
(2). SPA-IN-based local-global temporal representation
  A scattering-peak-aware instance normalization (SPA-IN) module is designed and embedded into the Conformer backbone. It fuses multi-scale pooled features to accurately capture the dominant scattering peaks and energy distribution of targets, and it leverages instance normalization to eliminate amplitude scale interference as well as clutter-induced feature aliasing and scattering information loss. Existing temporal Transformers generally adopt batch normalization and naive global average pooling, which tend to smooth out sparse strong scattering structures of targets. The proposed SPA-IN module simultaneously addresses two critical drawbacks: cross-domain statistical mixing and loss of scattering signatures.
(3). Reliability-aware fine-grained prototype calibration
  Global distribution alignment only mitigates overall domain bias and fails to eliminate category center offsets, which easily triggers negative transfer under few-shot settings. The proposed RAPC module dynamically constructs target-domain prototypes using a small number of labeled measured samples and guides cross-domain features to converge toward real measured category centers, compensating for the deficiency of global alignment that neglects fine-grained semantic offsets. Conventional prototype networks only build fixed prototypes based on source-domain simulated data and cannot adapt to feature manifold variations caused by measured clutter and attitude perturbations, whereas our method realizes dynamic updating of target-domain prototypes.
(4). Orthogonally regularized multi-sub-center metric optimization
  To tackle intra-class multimodal distribution and feature aliasing induced by variable attitudes and clutter interference, spherical angular margin loss is transplanted to 1D HRRP sequential tasks, with orthogonal constraints introduced to decouple multiple sub-centers. Multiple sub-centers are utilized to fit scattering manifolds under diverse attitudes, while orthogonal regularization enlarges inter-class angular margins to alleviate negative transfer and catastrophic forgetting, hence improving generalization in complex electromagnetic environments. Directly applying the original 2D-oriented sub-center ArcFace to one-dimensional time series leads to severe sub-center drift and feature overlap. The orthogonal regularization proposed in this work is a customized improvement tailored to the inherent characteristics of 1D HRRP sequences.

## 2. Related Work

Scarce measured HRRP samples, simulation-measurement distribution shift, and complex electromagnetic distortion are three persistent bottlenecks restricting the practical deployment of radar automatic target recognition (RATR). Extensive efforts have been devoted to cross-domain adaptation, few-shot learning, and robust temporal feature modeling for HRRP recognition in recent years, especially a series of hierarchical transformation frameworks proposed to strengthen cross-domain transferability in remote sensing and radar echo processing tasks [4,5,6]. However, most existing methods are tailored for two-dimensional optical/SAR images or fully labeled radar datasets, and they cannot simultaneously meet the demands of limited measured annotations, anti-clutter robustness, and simulation-to-real generalization under adversarial electromagnetic environments, leaving sufficient room for further exploration.

### 2.1. Cross-Domain Adaptation

Early HRRP recognition methods mainly relied on shallow models, such as SVM [7] and fuzzy matching [8], for classification based on handcrafted features [9,10], which lacked generalization ability and robustness against waveform distortion. With the advancement of deep learning, data-driven feature extraction has gradually replaced manual design. Guo et al. [11] utilized 1D-CNN to adaptively extract target scattering features and validated the effectiveness of deep networks for HRRP signal modeling. Xu et al. [12] further introduced recurrent networks to capture temporal dependencies of attitude-varying HRRP sequences. Nevertheless, these typical deep models require sufficient labeled data and suffer from severe performance degradation in few-shot measured scenarios.

Domain adaptation methods have been widely adopted to reduce simulation-measurement distribution gaps, mostly through adversarial feature alignment [13,14]. As a classic domain adaptation framework, DANN [15] leverages gradient reversal to implement adversarial domain learning, which has inspired numerous follow-up studies. Advanced strategies, including MMD, covariance matching, and contrastive learning, are further employed to alleviate domain discrepancy [16,17,18]. In recent remote sensing cross-domain research, hierarchical transformation composition schemes have been extensively studied to reinforce cross-domain transferability [4,5,19,20]. Wang et al. [4] proposed a hierarchical feature progressive alignment network that established coarse-to-fine multi-level mapping branches to unify feature spaces of multi-source remote sensing data. Zhu et al. [19] designed an invariant domain-level prototype memory module, which aggregated and decomposed category features layer by layer to narrow cross-domain distribution offsets. For cross-modal SAR detection tasks, HieraRS [5] constructed dual-branch hierarchical semantic transformation to realize knowledge transfer between optical and SAR images, while frequency-aware hierarchical prototype alignment [20] eliminated speckle noise layer by layer to improve generalization under multiple imaging conditions. However, all of these hierarchical frameworks were developed for two-dimensional remote sensing images and cannot fit the one-dimensional sequential scattering characteristics of HRRP echoes. More importantly, they only adopted flat single-layer hierarchical alignment without separate processing for global domain deviation and category-level center offsets. Under extremely scarce measured samples, global adversarial optimization and rigid distribution matching easily induce high computational overhead and negative transfer, where measured noise distorts simulated scattering priors [21]. To solve this problem, this paper abandons complex adversarial structures and adopts lightweight statistical moment matching combined with layer freezing and fine-tuning strategies to suppress few-shot negative transfer from the data level [22,23].

Most existing cross-domain strategies only implement single-layer global distribution matching and ignore the subsequent category-level offset correction. Our paradigm combines lightweight moment pre-alignment with later reliability-aware prototype calibration, forming a two-stage cross-domain correction pipeline to jointly suppress global domain shift and intra-class feature dispersion, which is essentially different from flat single-layer hierarchical transformation frameworks adopted in existing remote sensing works [4,5,20].

### 2.2. Few-Shot Learning

Few-shot learning can learn discriminative features from limited labeled samples, which matches the practical scenario of insufficient measured radar HRRP data. Existing few-shot solutions mainly include vision-language priors, scattering matching [24], and prototypical networks. In particular, Euclidean-distance-based prototypical networks have simple structures and are widely used for temporal recognition tasks.

**Figure 1:**
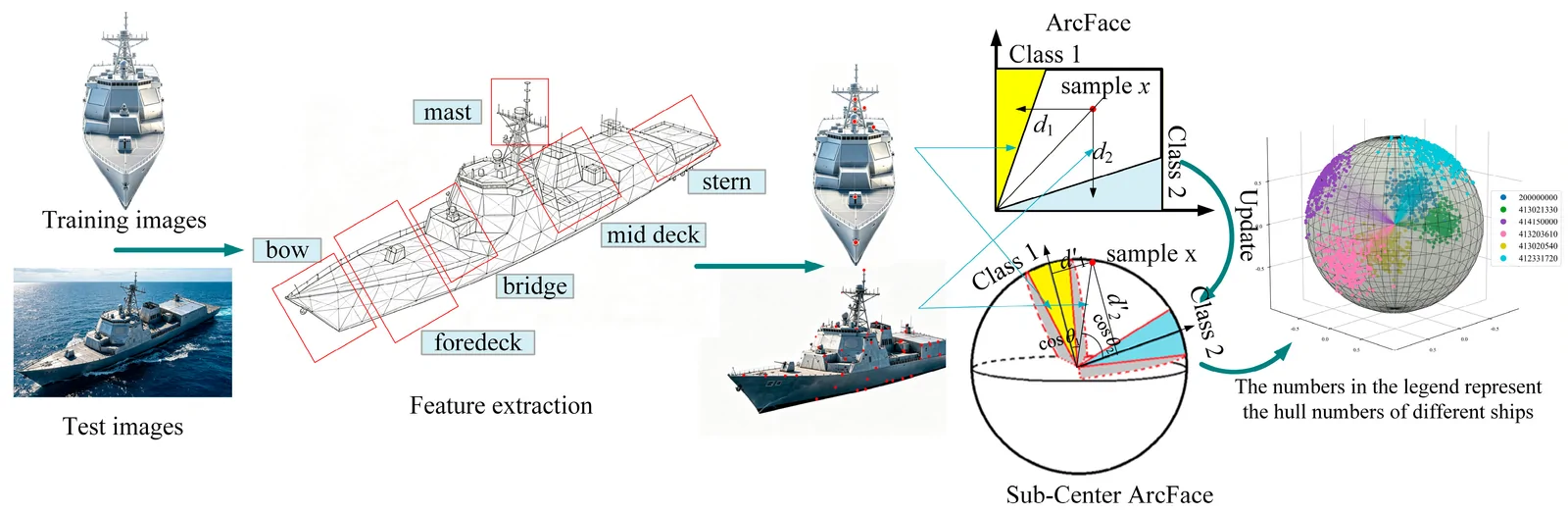
Classification examples of ArcFace and sub-center ArcFace.

Due to varying aspect angles and multipath effects, HRRP features of identical targets exhibit complex multimodal and non-convex distributions [25]. Single-prototype matching with Euclidean distance cannot fit such intricate scattering manifolds, easily causing intra-class feature confusion and blurred classification boundaries. In computer vision, angular metric learning methods such as ArcFace [26] and sub-center ArcFace [27] improve feature discrimination via additive angular margins and multi-sub-center structures (see Figure 1 for the principle), which are effective for multimodal image data and provide manifold optimization inspiration. However, these two-dimensional constraint mechanisms are incompatible with one-dimensional HRRP temporal signals, and direct application will cause sub-center drift and inter-class feature aliasing. Besides, existing hierarchical transfer learning for radar HRRP only conducts multi-scale time-spectrum decomposition without orthogonal decoupling constraints for intra-class multi-modal scattering manifolds [28].

Targeting the one-dimensional temporal and multi-angle scattering characteristics of HRRP, this paper improves the conventional 2D metric framework. By migrating angular margin constraints to HRRP temporal feature learning and introducing orthogonal regularization for sub-center decoupling, an orthogonal-constrained multi-sub-center ArcFace metric framework is proposed. Multiple sub-centers are used to fit scattering manifolds under different target attitudes, and orthogonal penalties suppress sub-center drift and feature overlap. This method effectively enhances cross-domain recognition robustness under few-shot and complex clutter conditions.

In summary, the existing angular metric frameworks are designed for two-dimensional spatial images and lack orthogonal decoupling constraints tailored to one-dimensional time-series scattering manifolds. The orthogonal multi-sub-center ArcFace proposed in this paper fills this gap for HRRP few-shot recognition.

### 2.3. Temporal Robustness Modeling

Clutter interference and partial echo occlusion seriously degrade HRRP recognition accuracy, requiring the network to extract local scattering details and global waveform topology simultaneously. 1D-CNN is lightweight and efficient for radar devices but suffers from limited receptive fields, failing to capture long-range temporal dependencies and yielding biased feature judgments for distorted waveforms [29].

Transformer models capture global temporal correlations through self-attention, while masked self-supervised learning enhances robustness to corrupted signals. Integrating convolution and self-attention, 1D-Conformer achieves joint local-global feature modeling and has become a mainstream backbone for temporal sequence recognition [30,31,32]. In radar HRRP recognition, hierarchical time-spectrum transformation and multi-layer attention mechanisms have been exploited to mitigate cross-aspect and cross-SNR domain discrepancy [6,28]. Yun et al. [28] built multi-scale asymmetric convolution with hierarchical time-spectrum decomposition to weaken attitude-induced waveform deviation. Bai et al. [6] introduced hierarchical multi-task sparse collaborative training for one-dimensional radar echo open-set identification. However, these hierarchical temporal learning schemes still relied on batch normalization and naive average pooling, which cannot separate cross-domain statistical bias and inevitably filter out sparse strong scattering peaks of targets.

Nevertheless, existing temporal Transformer variants adopt batch normalization, which is sensitive to echo amplitude and radar gain fluctuations. It mixes statistical characteristics of simulated and measured data during cross-domain fine-tuning, resulting in domain feature contamination [33]. Moreover, conventional channel attention adopts global average pooling, which smoothes sparse strong scattering peaks and background noise in HRRP, while losing critical target structural features.

To solve the above problems, this paper constructs a scattering-peak-aware instance normalization (SPA-IN) module embedded in the 1D-Conformer backbone. Instance normalization eliminates amplitude scale dependence and avoids cross-domain statistical mixing. Meanwhile, the designed peak-aware mechanism preserves key strong scattering information and avoids feature suppression [34], comprehensively improving the feature representation ability for distorted HRRP samples.

### 2.4. Cross-Domain Prototype Learning and Target-Domain Category Alignment

In radar HRRP cross-domain recognition, inherent distribution discrepancies between simulated and measured data severely degrade recognition performance. Most existing domain adaptation methods achieve global distribution alignment via moment matching, adversarial learning, or metric optimization, while ignoring fine-grained category center offsets. Under few-shot settings with sufficient simulated data and scarce measured annotations, pure global alignment only mitigates global domain bias but fails to eliminate semantic category-level deviation, easily causing negative transfer, intra-class feature dispersion, and ambiguous classification boundaries.

Prototype learning has become a mainstream solution for few-shot HRRP recognition. In remote sensing cross-domain research, numerous hierarchical transformation frameworks adopt multi-layer prototype aggregation and decomposition to enhance cross-domain transferability [5,19]. However, such methods only construct static prototype libraries based on source-domain optical or SAR images and cannot dynamically adjust category centers guided by limited labeled target-domain samples. Nevertheless, most existing methods build fixed source-domain prototypes [35] and cannot adapt to real-world feature manifold variations caused by clutter distortion, amplitude jitter, and target attitude perturbation. Although statistical prototype networks utilizing Mahalanobis distance [36] can relieve SNR-related domain mismatch, they still lack target-domain dynamic update ability.

Current radar domain adaptation methods rely heavily on pseudo labels and local MMD constraints [37], which suffer from poor robustness under extremely sparse labeled samples. Moreover, existing millimeter-wave radar cross-domain adaptation approaches focus on feature space unification but neglect specialized prototype calibration mechanisms for one-dimensional HRRP temporal scattering characteristics [38,39]. The comprehensive review on SAR cross-domain hierarchical learning further confirms that flat layered alignment without target-domain prototype updating leads to severe performance degradation under scarce labeled samples [40].

To address the above issues, this paper proposes a reliability-aware target-domain prototype calibration module. It dynamically updates target-domain category prototypes using limited measured samples to drive cross-domain features toward the real measured category SPA-IN centers. The proposed module compensates for the deficiency of traditional global alignment in correcting category-level domain offsets, providing robust fine-grained semantic constraints for few-shot cross-domain HRRP recognition.

Different from single global alignment or static source prototype methods, the two-stage pipeline combining global moment matching and target-domain dynamic prototype calibration proposed in this paper is the core differentiated design, in contrast to flat hierarchical transformation-based remote sensing cross-domain algorithms [4,5,40].

## 3. Algorithm Introduction

**Figure 2:**
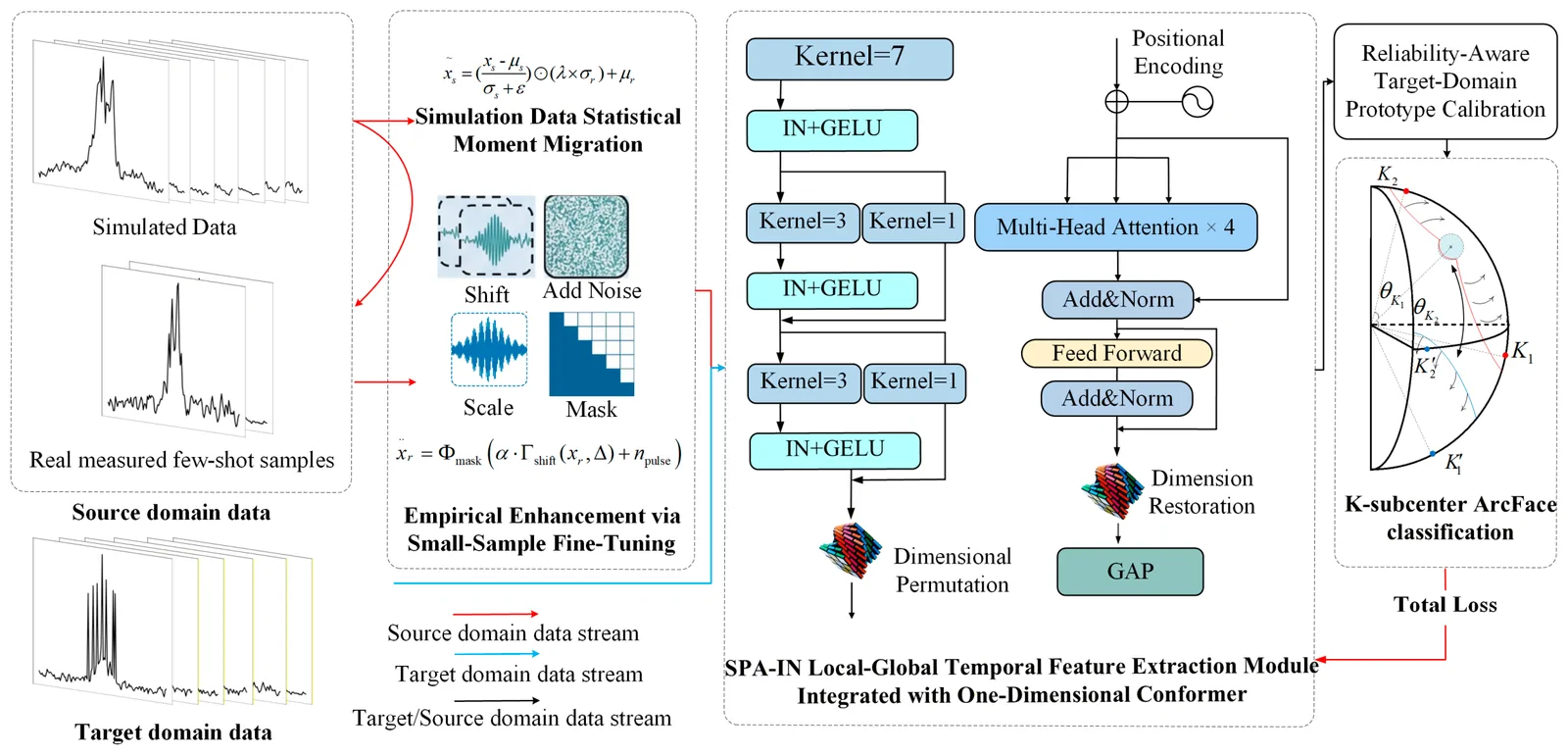
Flow chart of CROF-Net.

Aiming at the simulation-to-measurement cross-domain shift, limited labeled samples and complex battlefield electromagnetic interference in HRRP recognition, this paper proposes a cross-domain robust orthogonal few-shot network (CROF-Net). As shown in Figure 2, the framework extracts target scattering topological features via feature consistency constraints and adapts to three practical scenarios: pure echo, partial occlusion, and active jamming. It comprises four core modules: (1) cross-domain statistical feature alignment and robust data augmentation module; (2) SPA-IN-enhanced 1D Conformer module for local-global temporal feature extraction; (3) reliability-aware target-domain prototype calibration module; and (4) orthogonal regularization constrained sub-center ArcFace classification module.

### 3.1. Cross-Domain Feature Statistical Alignment and Robust Data Augmentation Module

Simulation-to-measurement cross-domain transfer suffers from inherent domain shift and overfitting caused by scarce measured samples. To tackle these issues, a dual-modal decoupled augmentation strategy is developed. For the simulation source domain, statistical moment alignment reduces global distribution discrepancies with measured data under consistent attitudes. For few-shot measured samples, diverse perturbation operations are adopted during training to enrich feature diversity and suppress fine-tuning overfitting. All input data are uniformly $L_{2}$-normalized to eliminate amplitude-scale interference and enhance the stability of cross-domain feature representation.

#### 3.1.1. Statistical Moment Transfer of Simulated Data

Suppose the dimension of a single HRRP sample in training is *L*. For simulated samples, the mean and standard deviation of the *i*-th dimension are denoted as $\mu_{i,s}$ and $\sigma_{i,s}$, respectively. Similarly, for few-shot measured HRRP samples, the mean and standard deviation of the *j*-th dimension are $\mu_{j,r}$ and $\sigma_{j,r}$, respectively. The simulated data are processed via a two-step serial transformation to fit the statistical characteristics of measured samples with the corresponding attitude. The value of the *i*-th dimension in the transformed single sample is expressed as follows:

(1) $${\tilde{x}}_{i,s}=(\frac{x_{i,s}-\mu_{i,s}}{\sigma_{i,s}+\varepsilon }\;)⊙(\lambda \times \sigma_{i,r})+\mu_{i,r}\;$$

where ⊙ denotes the Hadamard product, $\varepsilon =10^{-8}$ represents a numerical stability constant, and λ is the variance expansion coefficient used to moderately broaden the distribution range of corrected samples. Afterward, the samples ${\tilde{x}}_{s}$ are further $L_{2}$-normalized:

(2) $${\tilde{x}}_{s}=\frac{{\tilde{x}}_{s}}{\left\|{\tilde{x}}_{s}\right\|+\varepsilon }$$

**Figure 3:**
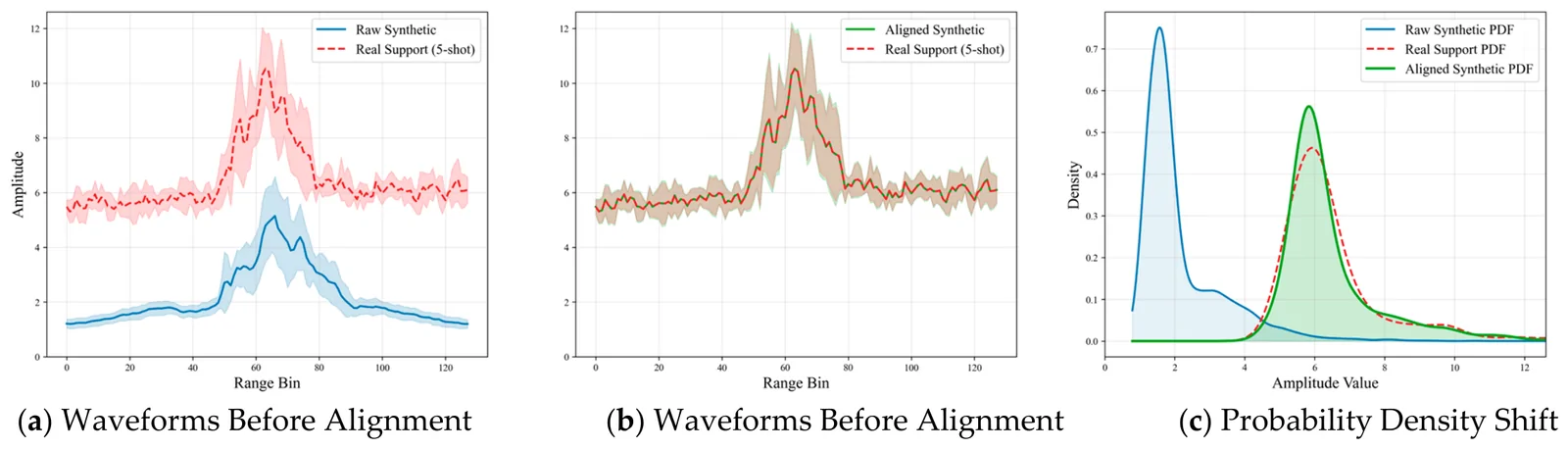
Visualization diagram of time-domain waveform envelope and amplitude probability density distribution of T72.

Taking the T72 target in SAMPLE dataset [41] as an example, each class uses 5 clean measured echoes for training (5-shot). Figure 3 compares time-domain envelopes and amplitude distributions before and after statistical moment alignment. The original simulated and measured waveforms differ greatly in baseline, variance, and time-domain features (Figure 3a). After alignment (Figure 3b), their mean trends and range cell fluctuations become consistent, with key scattering peaks well preserved. Kernel density curves (Figure 3c) show that the originally mismatched amplitude distributions achieve good agreement in peak position, range, and attenuation trend after calibration.

#### 3.1.2. Augmentation for Fine-Tuning with Few-Shot Measured Samples

During the fine-tuning phase, directly updating network weights using a small number of high-quality measured samples will lead to few-shot overfitting. To address this problem, we explore the global topological scattering correlation features of targets. Composite perturbation augmentation is introduced for measured samples before $L_{2}$ normalization in the fine-tuning process:

(3) $${\tilde{x}}_{j,r}=\Phi_{\mathrm{mask}}\left(\alpha \cdot \Gamma_{\mathrm{shift}}(x_{j,r},\Delta )+n_{\mathrm{pulse}\;}\right)\;$$

(4) $${\tilde{x}}_{r}=\frac{{\tilde{x}}_{r}}{\left\|{\tilde{x}}_{r}\right\|+\varepsilon }$$

Equation (3) indicates that each measured sample undergoes four cascaded physical transformations during training:

(1). Time delay jitter: Circular shift $\Gamma_{\mathrm{shift}}(x_{j,r},\Delta )$ is adopted to simulate the translation sensitivity of HRRP caused by gate center offset during actual radar tracking.

(2). Radar cross section (RCS) fluctuation: A scaling factor α is introduced to reproduce random RCS variations induced by target maneuvering or slight attitude changes.

(3). Additive pulse clutter injection: Since the HRRP envelope represents the absolute value of target scattering intensity, positive local spike mutation is adopted to test the robustness limit of the model. Positive pulses with specific amplitudes $n_{\mathrm{pulse}\;}$ are injected into randomly selected signal segments within the target region.

(4). Local scattering occlusion: A local mask $\Phi_{\mathrm{mask}}$ is applied. Consecutive range cells in the target area are randomly selected and set to zero, simulating local echo loss caused by stealth materials or physical occlusion.

With the combination of statistical moment transfer and fine-tuning augmentation, a manifold space adaptable to complex electromagnetic scenarios can be established using limited measured samples. In the simulation transfer stage, this strategy effectively compensates for distribution uncertainty caused by few-shot data. During measured data fine-tuning, it constrains the network to learn stable and essential global topological scattering correlation features of targets, thus mitigating the degradation of model generalization under extreme few-shot conditions from the perspective of feature representation.

### 3.2. Local-Global Temporal Feature Extraction Module Based on SPA-IN-Integrated 1D-Conformer

Considering the sensitivity of HRRP to local scattering features and its inherent long-range temporal dependence, this paper proposes a SPA-IN-embedded 1D-Conformer for temporal feature representation. Based on instance normalization, the network retains the relative variation of target scattering structures and mitigates cross-domain distribution shift. Furthermore, a scattering-peak-aware (SPA) mechanism is developed to optimize conventional channel attention. By integrating average and max pooling, the model simultaneously captures global RCS energy distribution and local strong scattering responses, thereby screening valid HRRP features and avoiding the loss of key scattering details. The network hierarchy, dimension variation, and core operations are summarized in Table 1.

### 3.3. Reliability-Aware Target-Domain Prototype Calibration Module

The SPA-IN-enhanced 1D-Conformer backbone yields distortion-insensitive temporal features, while global moment alignment cannot eliminate category center offset between simulation and measured domains. Under few-shot cross-domain settings, simulation-learned category centers deviate from the real measured distribution. To address this issue, this paper proposes a reliability-aware target domain prototype calibration module. It constructs category-wise weighted prototypes based on limited labeled measured samples and constrains cross-domain features to converge toward real measured category centers, achieving fine-grained category-level domain alignment.

#### 3.3.1. Target Domain Prototype Construction

The 1D-Conformer backbone outputs *D*-dimensional deep features $x\in R^{D}$. For the labeled measured dataset with *C* categories, the initial prototype of each category is generated by aggregating and averaging homogeneous measured sample features.

(5) $$p_{k}\;=\frac{1}{N_{r,k}}\;\;\sum_{x_{i\;}\in F_{r,k\;}}x_{i}\;\;$$

where $F_{r,k}\;$, $N_{r,k}$ represent the feature set and sample quantity of the *k*-th category in the measured domain, respectively. The confidence weight of each sample is calculated according to the distance between sample features and the initial prototype. Samples closer to the prototype are assigned higher weights. The robust target domain prototype is obtained via weighted fusion:

(6) $$p_{k}^{*}=\sum_{x_{i\;}\in F_{r,k\;}}w_{i,k}\;x_{i}$$

(7) $$w_{i,k}\;=\frac{\exp\left(-{\left\|x_{i}\;-p_{i}\right\|}_{2}\right)}{\sum_{x_{j}\in F_{r,k\;}}\exp\left(-{\left\|x_{j}-p_{k}\right\|}_{2}\right)}$$

All weights $w_{i,k}\;$ are normalized to suppress the negative impact of abnormal samples on category prototype construction.

#### 3.3.2. Prototype Constraint and Feature Calibration

After constructing target domain prototypes, a prototype alignment loss function is designed to constrain all sample features to gather around the target domain prototypes of their corresponding categories:

(8) $$L_{\mathrm{proto}\;}=\frac{\;1}{N_{\mathrm{all}}}\;\sum_{i=1}^{N_{\mathrm{all}}}{\|x_{i}\;-p_{y_{i\;}}^{*}\|}_{2}^{2}$$

where $N_{\mathrm{all}}$ denotes the total number of samples involved in loss calculation, and $p_{y_{i\;}}^{*}$ represents the weighted prototype corresponding to each sample. This loss function is jointly back-propagated with classification loss $L_{\mathrm{arc}}$, orthogonal regularization loss $L_{\mathrm{orth}}$ and domain consistency loss $L_{\mathrm{cons}}$. The joint optimization not only drives the features of the simulation domain to approach the category centers of the measured domain but also narrows the intra-class feature distance within the measured domain.

#### 3.3.3. Visualization Verification

**Figure 4:**
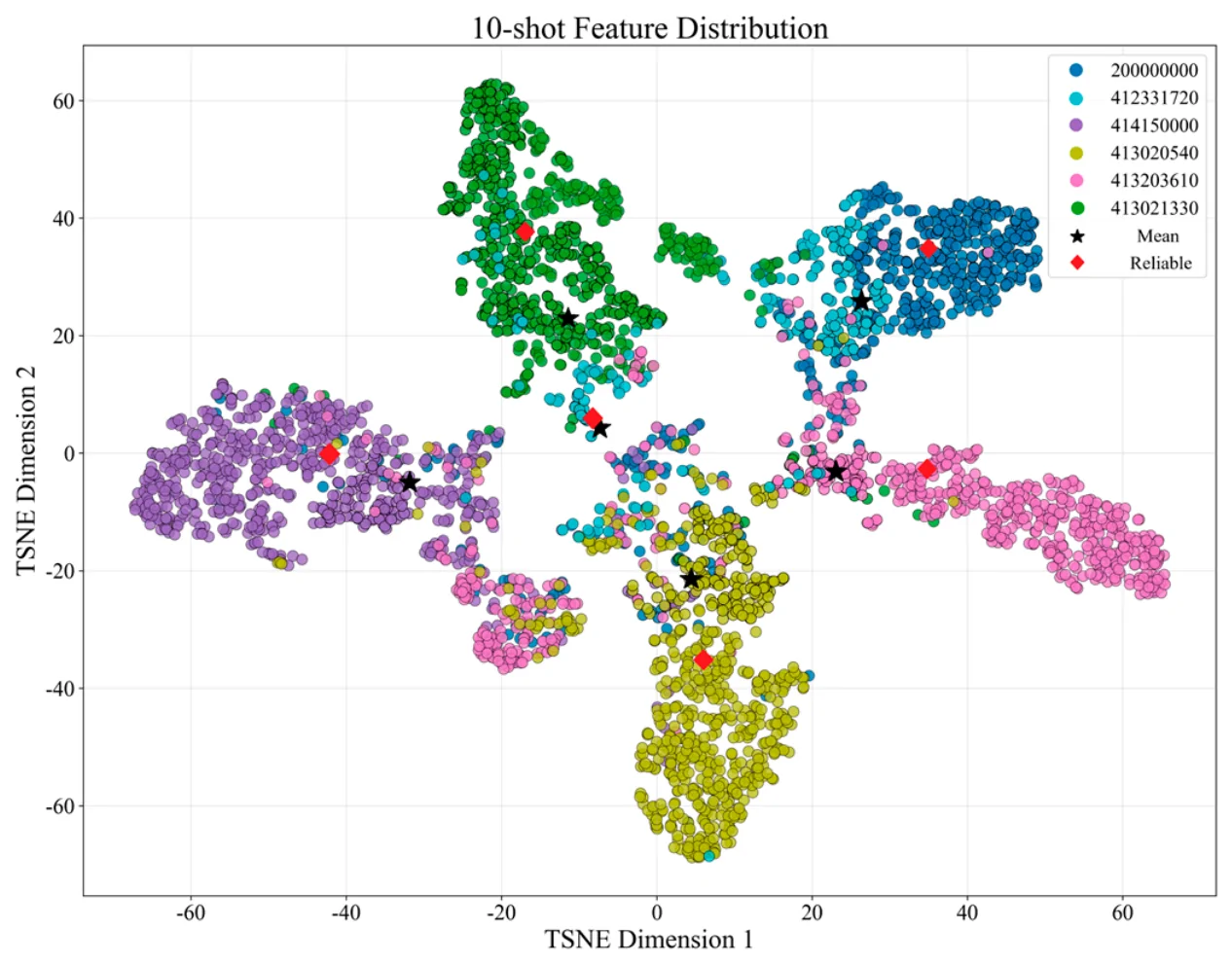
Visualization of t-SNE feature distribution with mean prototypes vs. reliability-aware prototypes under 10-shot cross-domain setting.

To intuitively validate the effectiveness of the proposed module, visualization comparisons are performed on the MTDSP dataset [35] under the 10-shot setting. Traditional mean prototypes and the proposed reliable prototypes are denoted by black pentagrams and red diamonds, respectively. The results shown in Figure 4 indicate that the traditional mean prototype method is susceptible to the interference of outlier noise, which leads to severe center shift. By contrast, the distance-weighted reliable prototypes suppress outlier interference and accurately locate in the dense core region of intra-class samples, fitting the real target-domain distribution centers well. This verifies that the reliability-aware prototype calibration module effectively corrects prototype deviation, enhances category center robustness, and provides stable metric constraints for few-shot cross-domain HRRP recognition.

### 3.4. Sub-Center ArcFace Classification Mechanism with Orthogonal Regularization Constraints

After prototype calibration corrects category offsets and compacts intra-class features, stable decision boundaries are required in the metric space. Different from 2D optical/SAR images with continuous spatial texture, radar HRRP is a one-dimensional electromagnetic scattering sequence, where strong scattering peaks shift drastically under varying target azimuth angles and multipath effects, forming complex intra-class multimodal scattering manifolds that cannot be well characterized by a single class centroid. Meanwhile, all HRRP features are normalized to unit hypersphere space in preprocessing, making hyperspherical angular measurement more robust than Euclidean distance against echo amplitude jitter, radar gain inconsistency, and simulation-measurement RCS mismatch. Targeting the unique characteristics of HRRP one-dimensional temporal signals, this paper proposes an orthogonal-constrained sub-center ArcFace classification mechanism. The multi-sub-center structure fits complex target scattering manifolds under varying attitudes and angular sectors, while the introduced orthogonal penalty enforces mutual repulsion among sub-centers. This strategy effectively suppresses disordered sub-center drift caused by few-shot learning and cross-domain deviation.

#### 3.4.1. Construction of Multi-Sub-Center Hyperspherical Angular Metric

Let the total number of target categories be *C*. To adapt to the typical scattering states of single targets in different azimuth sectors, we assign *K* learnable feature sub-centers to each category, and the global weight tensor $W\in R^{C\times K\times D}$ is defined accordingly. To eliminate interference from absolute amplitude caused by distance attenuation and RCS fluctuation, all sample features and sub-center weights are $L_{2}$-normalized uniformly. The similarity between samples and sub-centers is measured by cosine angle:

(9) $$\hat{x}=\frac{x}{{\left\|x\right\|}_{2}},\;\;{\hat{W}}_{c,k}=\frac{W_{c,k}}{{\left\|W_{c,k}\right\|}_{2}}\;$$

For any input sample with the ground-truth label $y_{i}$, we calculate the cosine similarity between the normalized feature $\hat{x}$ and all *K* sub-centers of the corresponding category. The maximum value is taken as the reference cosine value of the included angle for this category:

(10) $$\cos\theta_{y_{i}}=\max_{k\in \{1,\ldots ,K\}}({\hat{x}}^{T}\cdot {\hat{W}}_{y_{i},k})$$

This indicates that HRRP echoes acquired at different azimuth angles can adaptively match the nearest subcategory anchor on the corresponding manifold, which inherently addresses the dispersion of intra-class features caused by attitude sensitivity. Benefiting from the hyperspherical measurement paradigm, the model only focuses on the relative angular relationship between scattering features rather than absolute amplitude values, naturally resisting the cross-domain amplitude deviation between simulated and measured HRRP data.

On this basis, a scaling factor *s* (Set to 64) and an additive angular margin *m* are introduced to construct the sub-center ArcFace classification loss $L_{\mathrm{arc}}$ function tailored for HRRP few-shot scenarios:

(11) $$L_{\mathrm{arc}}=-\frac{1}{B}\sum_{i=1}^{B}\log\frac{e^{s\cdot \cos(\theta_{y_{i}}+m)}}{e^{s\cdot \cos(\theta_{y_{i}}+m)}+\sum_{j\neq y_{i}}^{C}\max_{k}(e^{s\cdot {\hat{x}}^{T}{\hat{W}}_{y_{i},k}})}\;$$

where *m* equivalently creates a buffer isolation zone for each target category in the hyperspherical space. For the cross-domain transfer task from simulated to measured data, it reserves fault tolerance for feature distribution shift during fine-tuning with measured samples and alleviates boundary ambiguity induced by domain discrepancy.

#### 3.4.2. Dynamic Sub-Center Orthogonal Regularization Constraint

Relying merely on multi-sub-center angular metrics cannot eliminate blurred feature boundaries under ultra-low signal-to-noise ratio and partial occlusion. If the included angles between sub-centers of different categories are too small on the hypersphere, severe noise disturbance will easily push sample features across decision boundaries and lead to misclassification. For one-dimensional HRRP signals with high feature dimension and scarce labeled measured samples, unconstrained multi-sub-centers tend to converge to similar directions under clutter interference, resulting in redundant scattering representation and sub-center drift. To tackle this problem, an explicit orthogonal penalty term $L_{\mathrm{orth}}$ is proposed to enforce orthogonality among the *K* sub-centers within each class on the hypersphere.

The three-dimensional global weight tensor W in Section 3.3.1 is reshaped into a two-dimensional sub-center weight matrix $\hat{W}\in R^{(C\cdot K)\times L}$, based on which the inner product similarity matrix of sub-centers $S=\hat{W}\cdot {\hat{W}}^{T}\in R^{\left(C\cdot K)\times (C\cdot K\right)}$ is calculated. A Boolean mask matrix Ω is then constructed, where the value is set to 1 for sub-centers from different categories and 0 for those within the same category. The constraint is only applied to subcenters of distinct targets, while penalties for intra-class sub-centers are masked. The final expression of the orthogonal loss is given as follows:

(12) $$L_{\mathrm{orth}}=\frac{1}{\left|\Omega \right|}\sum_{u}^{C\cdot K}\sum_{v}^{C\cdot K}\mathrm{ReLU}(S_{u,v}\cdot \Omega_{u,v}-\tau )$$

where τ denotes the orthogonal relaxation threshold. Combined with hyperspherical angular margin loss, this orthogonal constraint specially matches the multimodal scattering characteristic of one-dimensional HRRP: it decouples sub-centers to capture independent scattering modes under different target attitudes, eliminates redundant feature directions, and further strengthens the discriminability of 1D radar sequential features under few-shot cross-domain conditions. This mechanism enables the model to extract multi-dimensional, non-redundant, and robust discriminative features.

### 3.5. Loss Function

Combining the prototype alignment loss in Section 3.3, the orthogonal constrained multi-sub-center ArcFace classification loss, and the sub-center orthogonal regularization loss in Section 3.4, this paper further introduces a cross-domain consistency regularization constraint to construct a global joint loss function adapted to cross-domain few-shot recognition tasks. The loss function jointly achieves four optimization objectives: classification discrimination, sub-center decoupling, prototype calibration, and dual-domain feature alignment. The global loss is defined as follows:

(13) $$L_{\mathrm{total}}=L_{\mathrm{arc}}+\beta L_{\mathrm{orth}}+\gamma L_{\mathrm{cons}}$$

In the formula, β, γ, and δ are dynamic balancing coefficients used to adjust the weight of each loss term in joint optimization. $L_{\mathrm{cons}}$ represents the feature consistency constraint loss. It is designed to ensure that the deep topological semantics of simulated samples ${\tilde{x}}_{s}$ and the corresponding fine-tuned few-shot measured samples ${\tilde{x}}_{r}$ within the same batch remain highly consistent, and its calculation formula is given in Equation (14):

(14) $$L_{\mathrm{cons}}={\left\|{\tilde{x}}_{s}-{\tilde{x}}_{r}\right\|}_{2}^{2}$$

This joint loss function prevents the model from overfitting to superficial noise under the few-shot measured data setting and drives it to converge stably in the high-dimensional robust manifold space.

## 4. Experimental Results and Analysis

This section first introduces the datasets and unified experimental configurations. Multiple verification experiments are then conducted from various perspectives. The overall performance of the proposed CROF-Net is evaluated via recognition accuracy tests under different working conditions and comparisons with multiple baseline algorithms. Ablation experiments are carried out to verify the effectiveness of each module. Finally, hyperparameter sensitivity analysis is performed to fully demonstrate the rationality and robustness of the network design.

### 4.1. Dataset Configuration

#### 4.1.1. Ground Vehicle Dataset

The public SAMPLE dataset [41] is adopted for ground vehicle experiments. It contains simulated and measured SAR data of 10 vehicle targets under identical attitudes. In this work, the SAR images from the dataset are converted into one-dimensional HRRP sequences for experiments. The number of samples for each target is listed in Table 2.

To evaluate the anti-interference capability of the proposed algorithm under complex conditions, two challenging scenarios are constructed by augmenting the original measured samples:

(1). Additive pulsed Gaussian noise with an SNR of 5 dB is superimposed on target echoes to simulate discrete pulse electromagnetic interference;
(2). 30% of the core scattering area of each target is randomly occluded to emulate partial target blockage.

**Figure 5:**
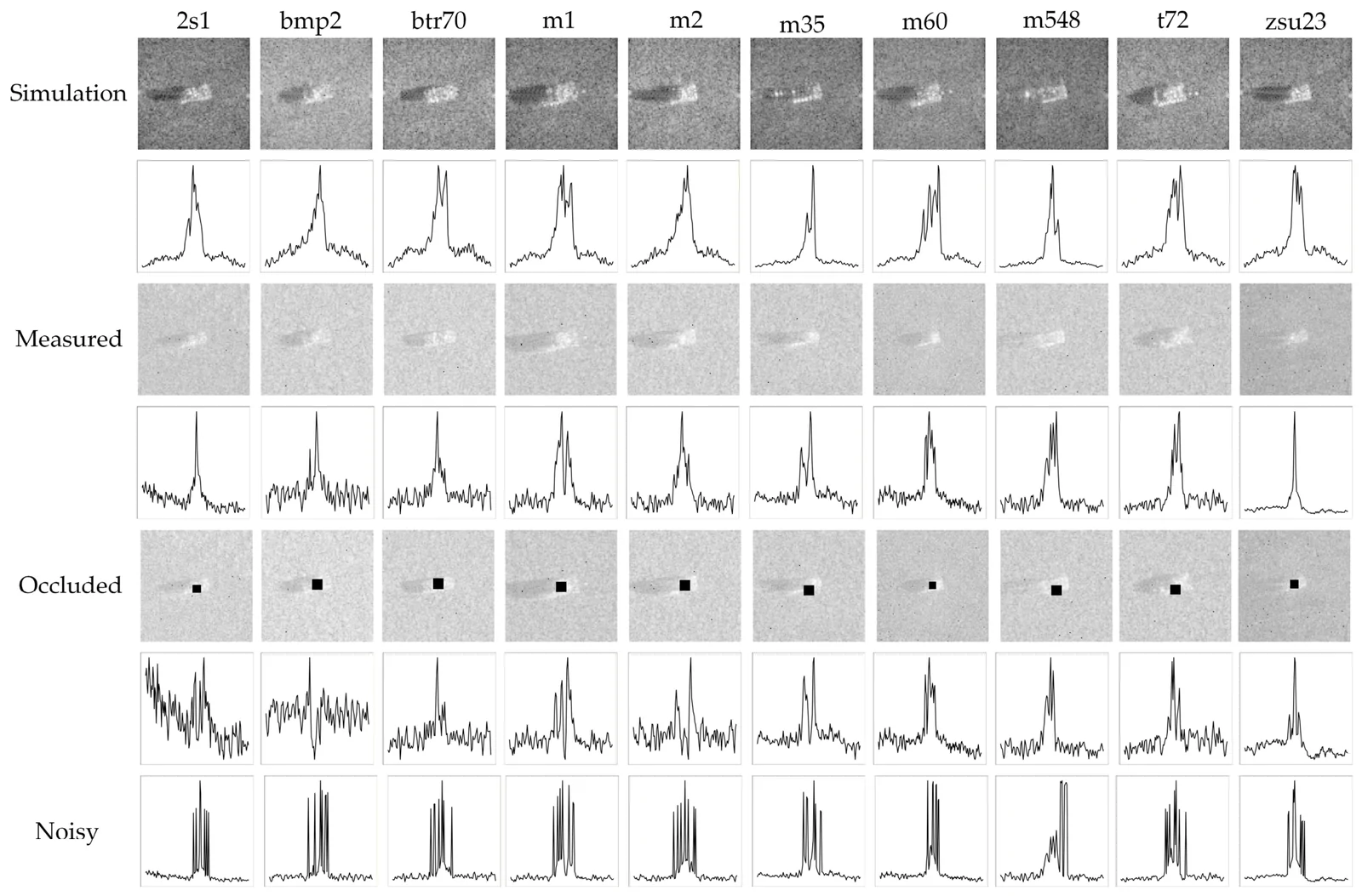
Scene condition settings for the SAMPLE dataset and corresponding sample examples of various types.

Figure 5 presents target categories of the SAMPLE dataset and typical HRRP waveforms under different working conditions.

#### 4.1.2. Sea Surface Ship Dataset

To fully validate the generalization ability of the proposed algorithm, experiments are further conducted on a sea surface ship HRRP dataset with more complex target structures, in addition to the SAMPLE vehicle dataset. The measured ship data are derived from the public MTDSP dataset [42], and echo samples of six maritime targets collected via VV polarization radar are selected. The auxiliary AIS information collected synchronously with each ship sample is listed in Table 3.

Corresponding simulated samples are generated using CST 2022 electromagnetic simulation software. Geometric models are established for the six selected ship targets, and the physical optics algorithm is adopted to calculate HRRP echoes. The hardware parameters of the simulated radar are completely consistent with those of the MTDSP measurement system. Simulated targets have a pitch angle of 0°–10° and a full azimuth range of 0°–360°.

**Figure 6:**
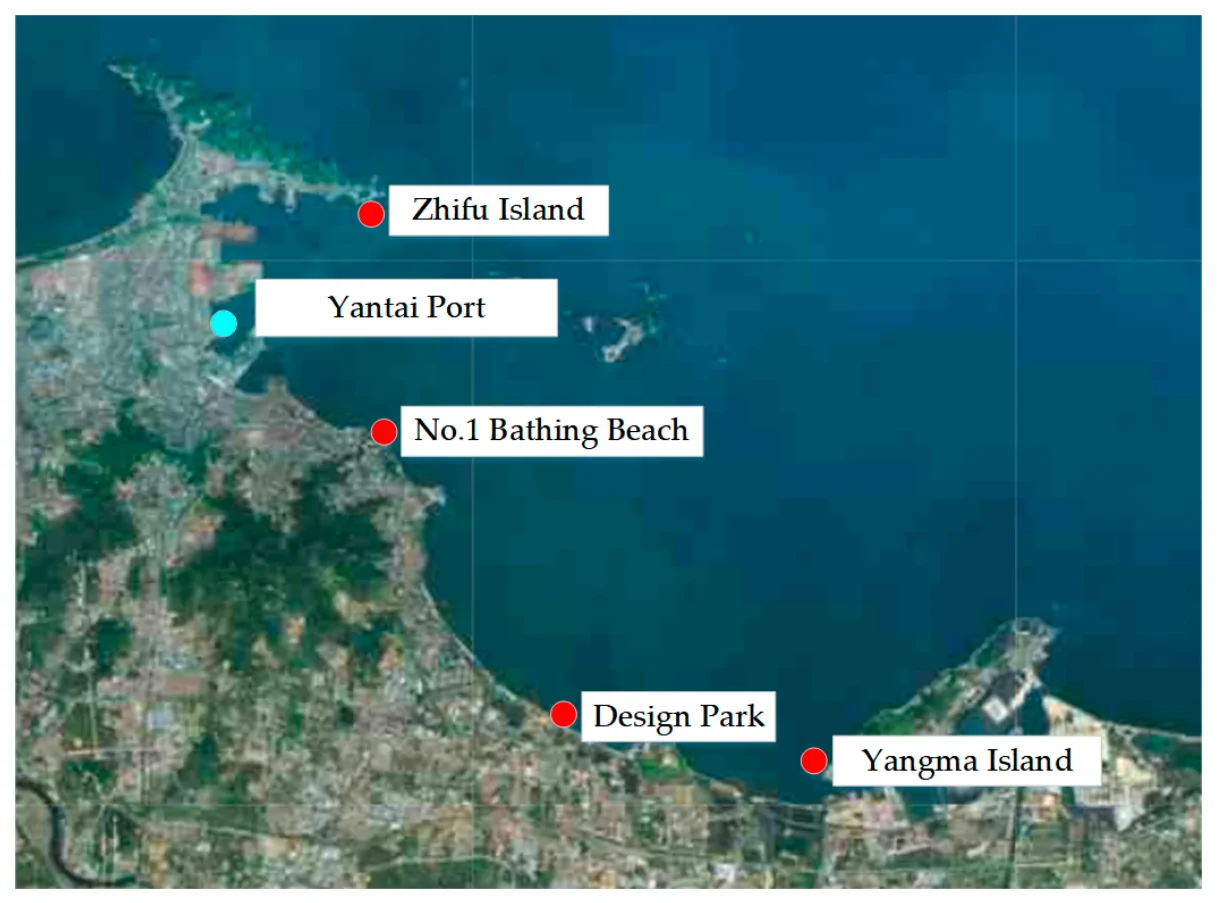
Set up radar observation points (red dots represent daytime observations, and blue dots represent nighttime observations).

The six ship categories in the MTDSP dataset have imbalanced measured samples. To reduce few-shot training overfitting, we conduct data augmentation on measured data. All radar and hardware parameters comply with the specifications in Ref. [43] and Table 4. The radar observation layout is shown in Figure 6.

**Figure 7:**
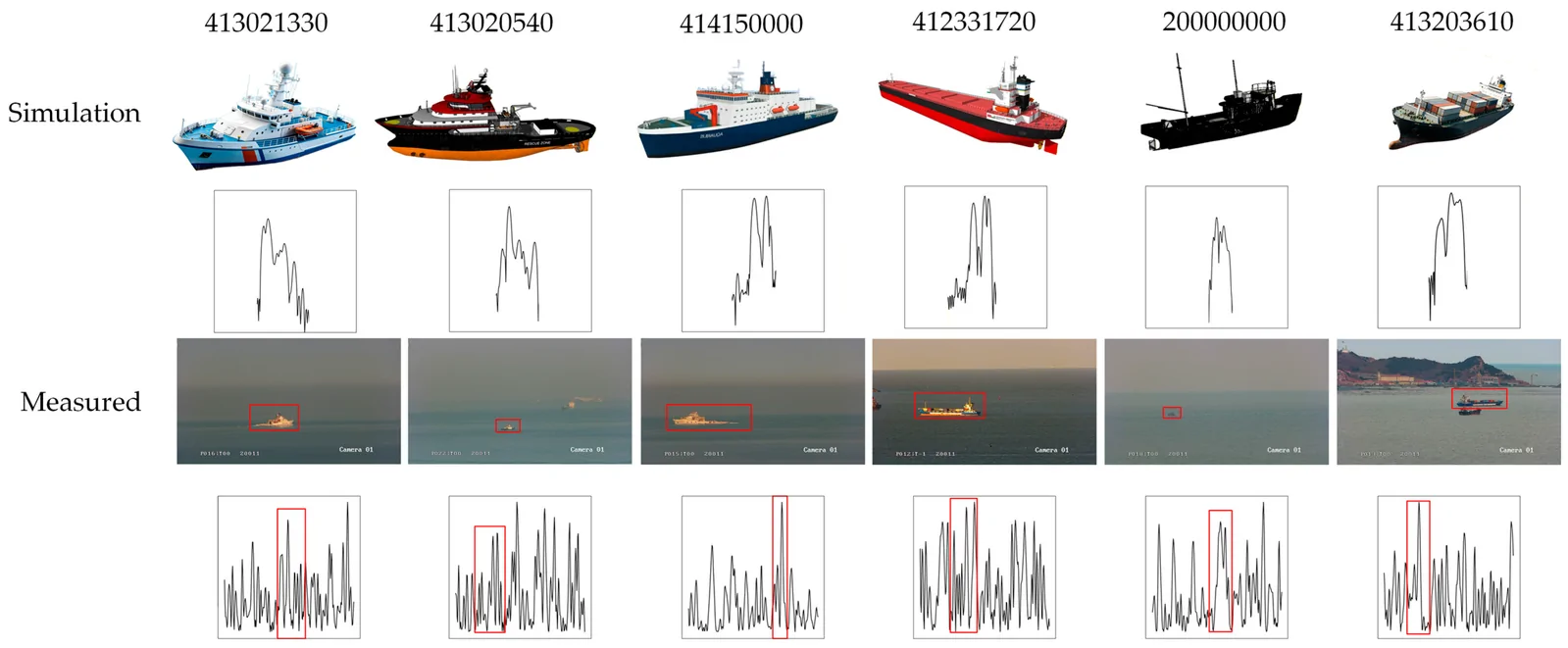
Optical images of ship targets and corresponding sample examples of various types (The red box represents the target echo in the real echo).

Restricted by variable marine conditions, only original simulated data and pure measured data are used for ship-related experiments, without additional artificial interference such as partial occlusion and impulse noise. Table 5 summarizes the sample sizes of the simulated training set, measured training set, and measured test set for each target category. Figure 7 shows the optical photos of ships under different sea conditions and the corresponding visualized HRRP waveforms.

### 4.2. Experimental Parameter Settings

The experiments are conducted on a workstation equipped with an Intel(R) Xeon(R) W-2295 CPU, 128 GB memory, and an NVIDIA RTX 5090 graphics card, running the Windows 10 operating system. Unless otherwise specified, the training hyperparameters are set as follows: total training epochs = 200, batch size = 32, and lr=0.0015. When simulated data are not involved in training, the dataset is split into a training set and a test set at a ratio of 8:2.

For the SAMPLE dataset, the hyperparameter setting is: number of sub-centers *K* = 1. For the MTDSP dataset, the hyperparameter combination is: number of sub-centers *K* = 5, orthogonal penalty coefficient β = 1.0, consistency constraint loss coefficient γ = 0.5, prototype alignment loss coefficient δ = 1.0, and angular margin *m* = 0.3. The term “shot" denotes the number of labeled measured samples per class used for model training.

### 4.3. Evaluation Metrics

To comprehensively evaluate the recognition accuracy, cross-domain transfer capability, feature representation quality, and practical deployment performance of the model, five quantitative metrics are adopted: recognition accuracy, feature shift, intra-class feature similarity, parameter count, floating point operations (FLOPs), and inference latency.

The feature shift $D_{\mathrm{shift}\;}$ is calculated as follows:

(15) $$D_{\mathrm{shift}\;}={\left\|\mu_{S}-\mu_{T}\right\|}_{2}$$

where $\mu_{S}=\frac{1}{N_{S}}\sum_{i=1}^{N_{S}}x_{S,i}$ and $\mu_{T}=\frac{1}{N_{T}}\sum_{j=1}^{N_{T}}x_{T,j}$ represent the mean feature vectors of all samples in the training set and test set, respectively. A smaller value indicates better domain alignment and stronger cross-domain generalization ability.

The intra-class feature similarity $S_{\mathrm{intra}}$ is defined as the average similarity of deep feature vectors within each class:

(16) $$S_{\mathrm{intra}}\;=\frac{2}{N_{T}(N_{T}-1)}\;\sum_{a=1}^{N_{T}-1\;}N_{T}\sum_{b=a+1}^{N}\frac{x_{a}^{T}x_{b}}{{\left\|x_{a}\right\|}_{2}{\left\|x_{b}\right\|}_{2}}\;\;\;\in [-1,1]$$

where $N_{T}$ be the total number of samples in a certain class of the test set, and $x_{a}$, $x_{b}$ denote two arbitrary deep feature vectors from this class. A value closer to 1 means higher model robustness.

### 4.4. Analysis of Recognition Accuracy

#### 4.4.1. Results on Vehicle Dataset

**Figure 8:**
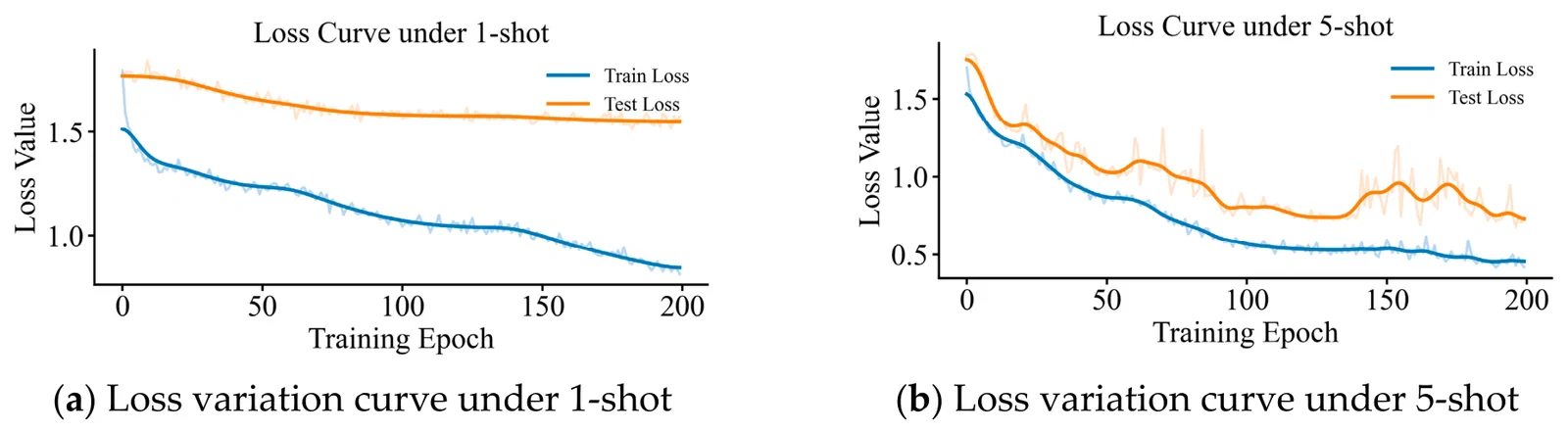

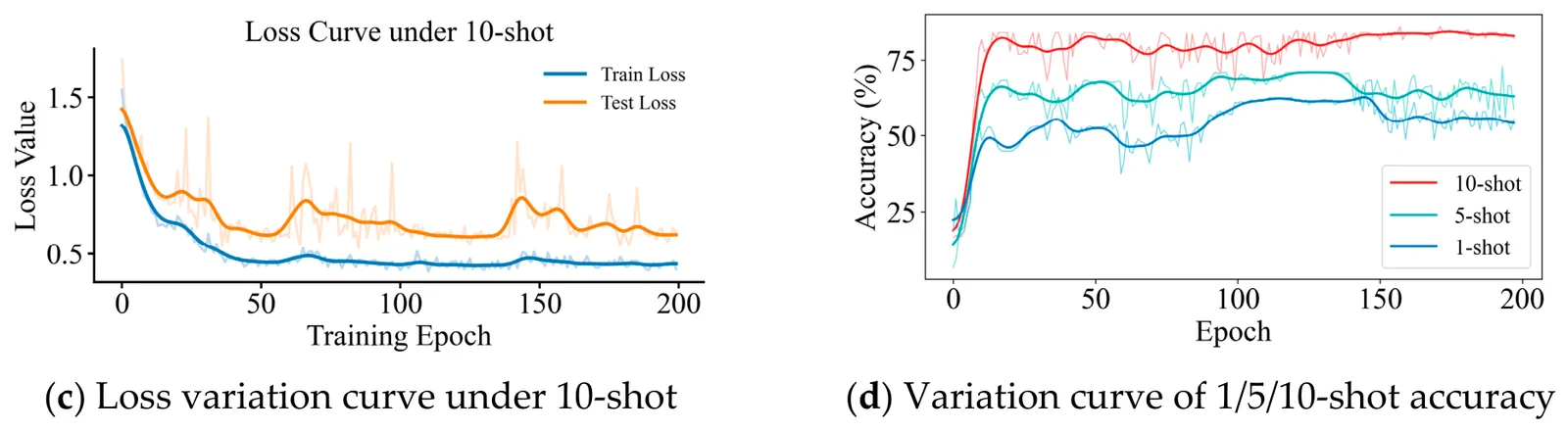
Loss and accuracy variation curves of the simulation plus actual measurement fine-tuning method (SAMPLE).

**Figure 9:**
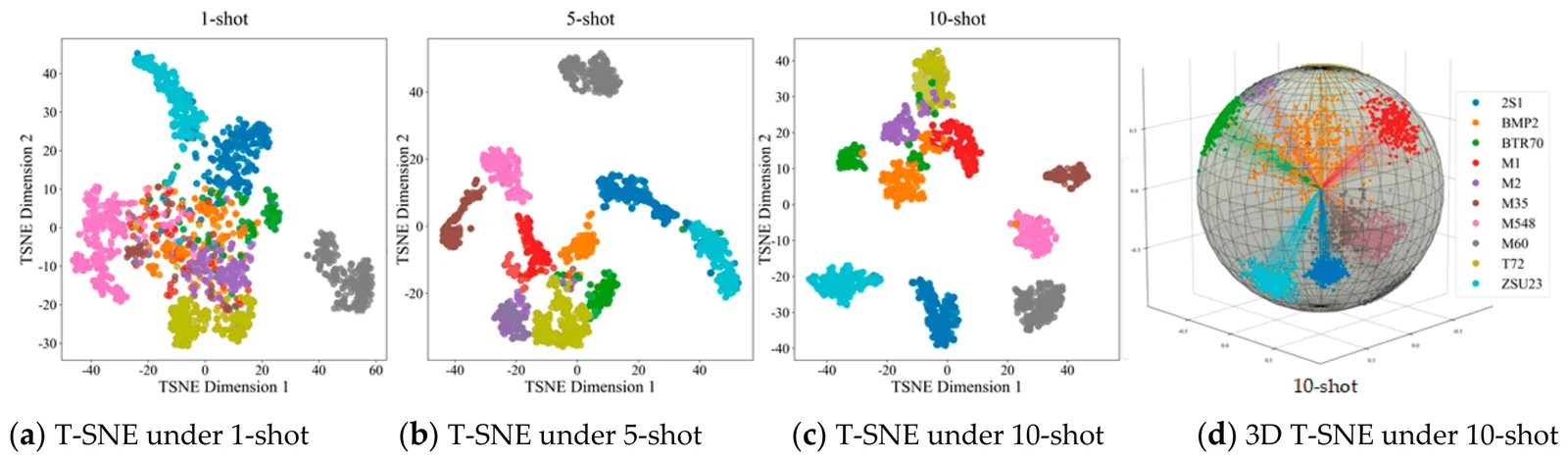
T-SNE visualization of simulation + measured data fine-tuning method (SAMPLE).

**Figure 10:**
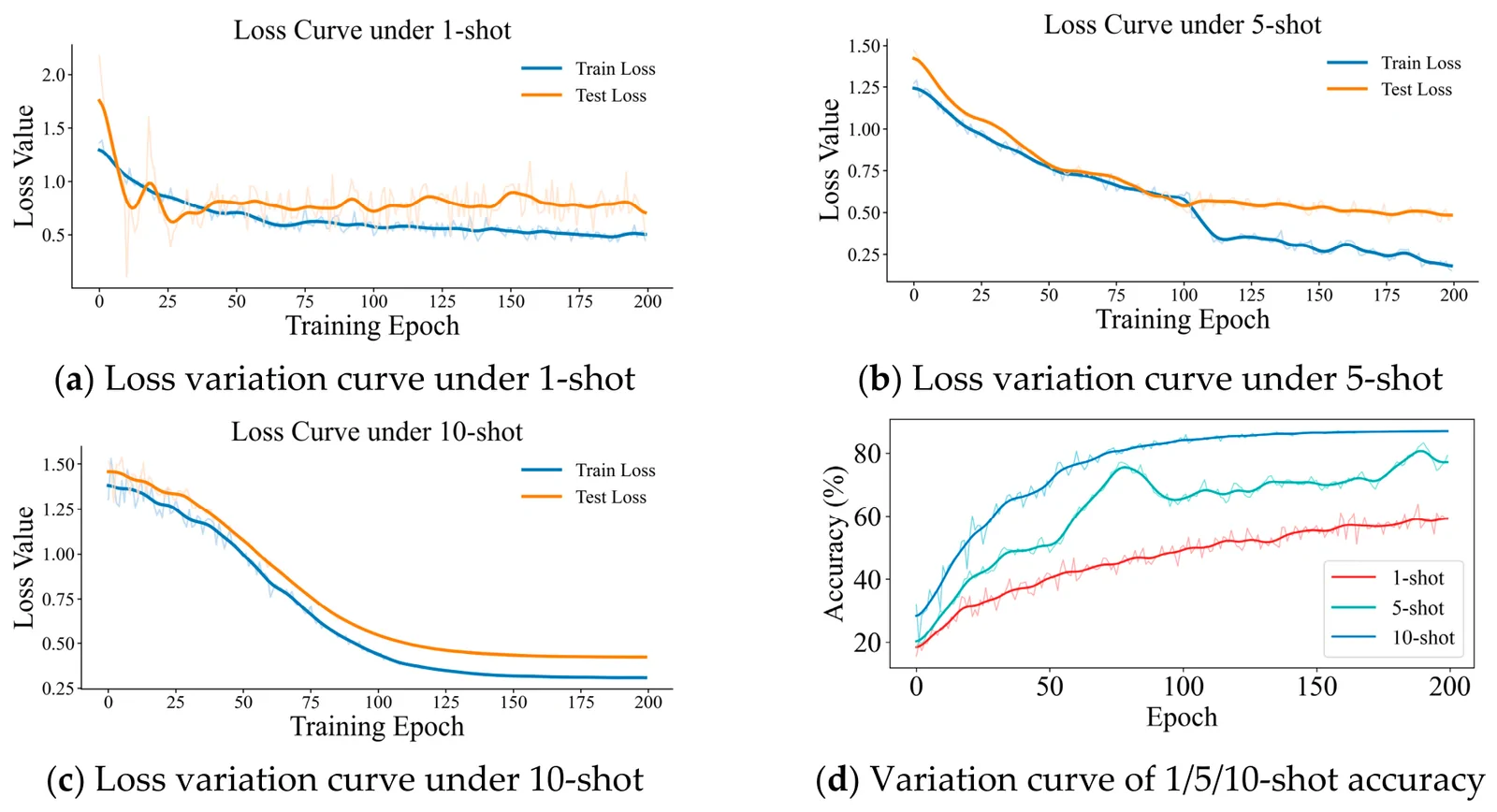
Loss and accuracy variation curves of the simulation plus actual measurement fine-tuning method (MTDSP).

**Figure 11:**
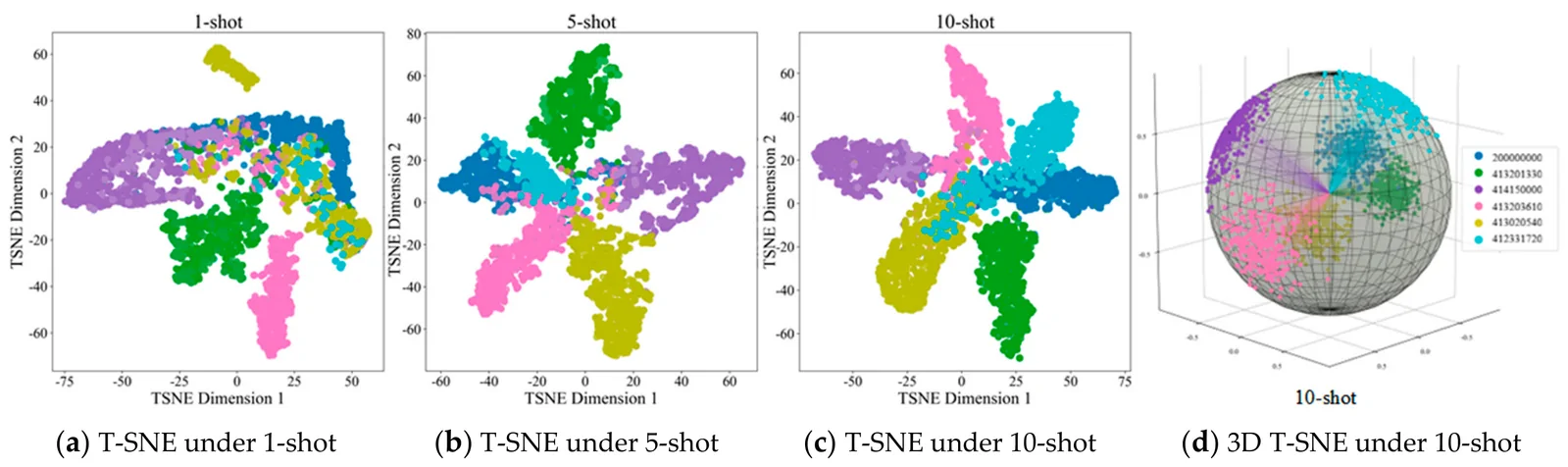
T-SNE visualization of simulation + measured data fine-tuning method (MTDSP).

To verify CROF-Net’s anti-interference and generalization abilities, we adopt three training schemes: purely simulated data, purely measured data, and simulated data plus augmented samples. Tests are conducted under three scenarios: clean echoes, partial occlusion, and pulse interference. Results from purely simulated and purely measured training serve as performance bounds. We analyze the model’s strengths and limitations, with the quantitative results shown in Figure 8, Figure 9, Figure 10 and Figure 11.

(1). Basic scenario: Recognition accuracy under purely measured echo condition

Models fine-tuned only on scarce measured samples face limited feature optimization. As a domain adaptive framework trained jointly on simulation and measured data, CROF-Net obtains higher accuracy under few-shot conditions. With more labeled samples, its accuracy grows steadily (Table 6) and converges well (Figure 8). t-SNE results (Figure 9) also show tighter intra-class clustering and stronger inter-class separation.

CROF-Net outperforms methods using measured data alone. In particular, combining simulated data with 10-shot measured samples delivers performance comparable to using full measured data. It effectively leverages scattering characteristics from the simulation domain, improves sample utilization, and relieves overfitting and catastrophic forgetting. The proposed method also achieves faster convergence and better cross-domain generalization than single-domain fine-tuning.

(2). Information missing scenario: Analysis of model recognition robustness under occlusion

In Table 7, models trained with purely simulated data and fully occluded data serve as the lower and upper bounds, respectively. Large distribution gaps exist between clean and occluded measured samples. Single-domain few-shot training obtains low accuracy and poor generalization.

Two joint fine-tuning schemes are adopted. The model trained on simulation plus clean measured data is slightly less accurate than that using simulation and occluded data, yet it outperforms all single-domain methods. It can extract universal target scattering features to remedy feature defects caused by occlusion. The combination of simulated and occluded data achieves the best performance.

Simulated data provide complete scattering topology priors to supplement limited occluded samples. With more labeled samples, the model reconstructs full features from valid scattering fragments, and its accuracy gradually approaches the upper bound. The results prove strong robustness against partial signal loss.

(3). Severe interference scenario: Accuracy and robustness analysis under noisy conditions

In Table 8, purely simulated data and fully noisy data correspond to the lower and upper performance bounds. Obvious domain shift exists between clean and noisy measured samples. Single-domain few-shot models show weak generalization and anti-interference abilities with low accuracy.

The performance of the proposed model trained on simulation plus clean measured data is slightly inferior to that of its simulation-noisy counterpart but still surpasses all single-domain methods. It captures inherent scattering characteristics to mitigate noise interference. The simulation-noisy joint training achieves the best results.

Clean simulated data offer reliable scattering priors, prevent the model from overfitting to noise, and compensate for insufficient valid features in few-shot learning. Accuracy rises steadily with more labeled samples and approaches the upper limit.

Notably, CROF-Net focuses on local scattering feature extraction for occlusion and noise resistance, which makes it somewhat sensitive to impulse noise, forming an inherent performance trade-off.

#### 4.4.2. Analysis of Recognition Results for Ship Targets

Different from vehicle HRRP, ship echoes are disturbed by sea clutter, multipath effects, and structural occlusion. We use real ship measured data to evaluate the model’s generalization in complex marine environments (Table 9, Figure 10 and Figure 11).

Large domain shift makes purely simulation-based cross-domain transfer ineffective. The full measured dataset sets the performance upper bound, while few-shot single-domain fine-tuning easily causes overfitting and feature scattering.

Experiments show that CROF-Net alleviates feature shift, optimizes feature clustering, and suppresses overfitting. Its performance improves steadily with increasing measured samples. Compared to vehicle recognition, the model delivers better robustness and convergence for ship targets in complex marine scenarios.

### 4.5. Comparative Experiments and Comprehensive Performance Analysis

To evaluate the overall performance of CROF-Net in few-shot cross-domain HRRP recognition, five representative mainstream algorithms are selected as baselines, including Scattering [44], SimCNN [45], TSQDA [46], GDBN-MAML [47], and TSML [48].

#### 4.5.1. Comprehensive Comparison on the SAMPLE Dataset (Table 10)

CROF-Net performs well under all few-shot settings. Its accuracy hits 62.32% in the 1-shot case, on par with GDBN-MAML. For the 5-shot and 10-shot settings, the accuracy increases to 72.82% and 84.10%, respectively, evidently outperforming GDBN-MAML, SimCNN, and other competitors. Its feature shift declines steadily with more samples and stays the lowest, confirming strong cross-domain alignment and intra-class aggregation ability.

**Table 10:** Recognition performance of different models with various few-shot sizes on the SAMPLE dataset (The bold values represent the optimal results).

Algorithm	Shot	Accuracy (%)	$D_{\mathrm{shift}\;}$ (↓)	$S_{\mathrm{intra}}$ (↑)	Params (M)	FLOPs (M)	Inference Time (ms)
GDBN-MAML	1	61.13	1.47	0.51	0.75	28.72	1.76
TSML		48.16	1.93	0.42	0.72	26.35	1.69
Scattering		47.79	1.98	0.40	0.81	30.15	1.88
SimCNN		51.69	1.91	0.45	**0.59**	**20.36**	**1.36**
TSQDA		52.62	1.85	0.48	0.78	29.64	1.82
**CROF-Net**		**62.32**	**1.42**	**0.53**	0.68	24.48	1.44
GDBN-MAML	5	65.87	1.29	0.56	0.75	28.72	1.75
TSML		62.16	1.43	0.53	0.72	26.35	1.69
Scattering		51.74	1.89	0.45	0.81	30.15	1.88
SimCNN		66.55	1.17	0.56	**0.59**	**20.36**	**1.36**
TSQDA		55.15	1.66	0.48	0.78	29.64	1.82
**CROF-Net**		**72.82**	**1.05**	**0.62**	0.68	24.48	1.44
GDBN-MAML	10	72.53	1.06	0.61	0.75	28.72	1.76
TSML		70.12	1.08	0.59	0.72	26.35	1.70
Scattering		55.10	1.68	0.48	0.81	30.16	1.89
SimCNN		74.41	0.97	0.63	**0.59**	**20.36**	**1.37**
TSQDA		57.39	1.61	0.49	0.78	29.67	1.87
**CROF-Net**		**84.10**	**0.65**	**0.71**	0.68	24.48	1.43

With 0.68 M parameters, 24.48 M FLOPs, and 1.44 ms inference latency, CROF-Net is lightweight. It achieves competitive accuracy and strikes a better balance between performance and efficiency than larger models like GDBN-MAML and TSQDA. The results validate its effectiveness and generalization for vehicle HRRP recognition.

#### 4.5.2. Comprehensive Comparison on the MTDSP Dataset (Table 11)

CROF-Net outperforms all competitors across all few-shot settings. It attains 63.91% accuracy under 1-shot and further improves to 83.36% (5-shot) and 87.20% (10-shot) with growing performance advantages. It consistently has the smallest feature shift and highest intra-class similarity, showing strong cross-domain alignment and feature aggregation.

With moderate parameters and computation cost, CROF-Net achieves a better accuracy-efficiency trade-off than costly models like GDBN-MAML and Scattering. Satisfactory results on the ship dataset with complex targets and harsh interference also verify its strong generalization for diverse scattering characteristics.

**Table 11:** Recognition performance of different models with various few-shot sizes on the MTDSP dataset (The bold values represent the optimal results).

Algorithm	Shot	Accuracy (%)	$D_{\mathrm{shift}\;}$ (↓)	$S_{\mathrm{intra}}$ (↑)	Params (M)	FLOPs (M)	Inference Time (ms)
GDBN-MAML	1	61.13	5.37	0.57	0.75	28.72	1.76
TSML		63.14	4.67	0.60	0.72	26.35	1.69
Scattering		62.74	4.83	0.58	0.81	30.15	1.92
SimCNN		48.14	7.11	0.41	**0.59**	**20.36**	**1.37**
TSQDA		32.75	9.62	0.21	0.78	29.64	1.88
CROF-Net		**63.91**	**4.5** **2**	**0.61**	0.68	24.48	1.43
GDBN-MAML	5	79.34	2.43	0.81	0.75	28.71	1.75
TSML		80.34	1.94	0.82	0.72	26.35	1.69
Scattering		81.54	1.67	0.83	0.81	30.15	1.92
SimCNN		54.41	6.50	0.49	**0.60**	**20.37**	**1.37**
TSQDA		38.62	8.66	0.29	0.78	29.64	1.89
CROF-Net		**83.36**	**0.96**	**0.86**	0.68	24.48	1.43
GDBN-MAML	10	83.14	1.04	0.86	0.75	28.73	1.77
TSML		81.09	1.81	0.83	0.72	26.35	1.69
Scattering		82.92	1.35	0.85	0.81	30.15	1.94
SimCNN		56.55	5.74	0.52	**0.59**	**20.36**	**1.37**
TSQDA		42.98	7.95	0.34	0.78	29.70	1.88
CROF-Net		**87.20**	**0.** **23**	**0.** **93**	0.68	24.48	1.45

With scarce measured samples, conventional deep models suffer from over-parameterization and feature degradation, performing worse than traditional machine learning methods in purely measured few-shot tasks. When combined with simulated data, traditional machine learning approaches fail to separate domains and incur severe negative transfer. Classic domain adaptation methods like DANN alleviate domain gaps but damage local high-frequency scattering features, leading to limited improvement for HRRP recognition. By contrast, CROF-Net adopts low-order moment matching and scattering-peak-aware design to avoid negative transfer, achieving prominent performance gains via joint training on simulated and measured data.

### 4.6. Baseline Comparison

To comprehensively evaluate the recognition performance of CROF-Net, this section constructs 9 baseline models covering traditional methods, standard deep learning models, and state-of-the-art few-shot/domain adaptation algorithms for thorough comparison, aiming to verify the stability and generalization ability of CROF-Net under the simulation-measurement joint training strategy. The results are presented in Table 12.

Under the constraint of extremely limited measured samples, conventional deep learning methods are prone to feature degradation due to over-parameterization, resulting in generally low accuracy in the purely measured few-shot scenario, with performance significantly inferior to that of traditional machine learning methods. When simulated data are introduced to enrich prior knowledge, traditional machine learning algorithms suffer from obvious negative transfer due to the lack of domain decoupling capability. Although classic domain adaptation methods such as DANN can partially mitigate domain discrepancy and achieve moderate performance gains, their global mandatory alignment mechanism tends to destroy the local high-frequency scattering topologies of radar signals, leading to limited improvements and poor adaptability to tasks highly sensitive to local scattering characteristics, such as HRRP recognition. By contrast, the proposed CROF-Net effectively blocks the negative transfer path through low-order statistical moment alignment and scattering-peak-aware mechanisms, achieving remarkable performance gains under the simulation-measurement joint training scenario.

### 4.7. Module Ablation Experiments

To quantitatively verify the individual contribution of each core module and their synergistic optimization effects, a progressive ablation study is conducted on the MTDSP dataset. The baseline model is a basic one-dimensional convolutional neural network (1D-CNN) without any proposed improvements. By incrementally adding each proposed component, the recognition accuracy under 1-shot, 5-shot, and 10-shot few-shot settings is compared to validate the necessity of each module. The results in Table 13 show that all modules consistently provide positive performance gains.

The layered ablation results in Table 13 demonstrate that all proposed modules consistently yield stable and positive performance gains under different few-shot scenarios, and the overall framework achieves significant cumulative optimization through multi-module synergistic coupling.

The detailed ablation analysis is illustrated as follows: (1) Compared to the baseline, the EDA-Aug data augmentation strategy (G1) achieves a substantial accuracy improvement under ultra-low 1-shot conditions. It effectively compensates for the scarcity of measured HRRP samples and constructs robust pre-training priors, alleviating the overfitting problem caused by insufficient labeled radar data. (2) The embedded SPA-IN module (G2) further improves recognition accuracy by a large margin. By eliminating cross-domain statistical mixing caused by batch normalization and preserving sparse strong scattering peak features of HRRP echoes, the module enhances the anti-interference ability of temporal feature representation under complex clutter backgrounds. (3) The introduced OMS-ArcFace metric learning module (G3) brings continuous performance improvement. Benefiting from the hyperspherical angular margin mechanism adapted to normalized 1D radar scattering features and the orthogonal decoupling constraint for multiple sub-centers, this module accurately fits the intra-class multimodal scattering manifold caused by target attitude changes, suppresses sub-center drift and feature aliasing in few-shot cross-domain scenarios, and solves the limitation that single-center Euclidean metric and ordinary 2D angular metric cannot adapt to HRRP one-dimensional sequential characteristics. (4) The full CROF-Net framework integrates the RAPC fine-grained prototype calibration module, which corrects the category-level center offset ignored by global domain alignment. Combining data-level pre-alignment, feature-level robust modeling, semantic-level prototype optimization, and metric-level discrimination enhancement, the proposed method realizes end-to-end cross-domain robust optimization and achieves the optimal recognition accuracy in all few-shot settings.

In summary, each core module of CROF-Net targets the unique practical bottlenecks of radar HRRP few-shot cross-domain recognition, and the progressive ablation results fully validate the independent effectiveness and excellent synergistic complementary performance of all components.

### 4.8. Parameter Optimization Experiments

To explore the influence of network hyperparameters on the recognition performance of different radar targets, systematic parameter ablation experiments are conducted on the SAMPLE and MTDSP datasets under the simulation-assisted 10-shot cross-domain setting. Five key hyperparameters are investigated, including the number of sub-centers *K*, orthogonal penalty coefficient β, consistency constraint coefficient γ, prototype alignment loss weight δ, and angular margin *m*. All hyperparameter values selected in the final CROF-Net are determined based on both quantitative sensitivity experimental results and inherent electromagnetic scattering laws of radar HRRP targets, rather than empirical random settings. We further analyze why different targets correspond to distinct optimal hyperparameter configurations from the perspective of target scattering distribution characteristics.

**Figure 12:**
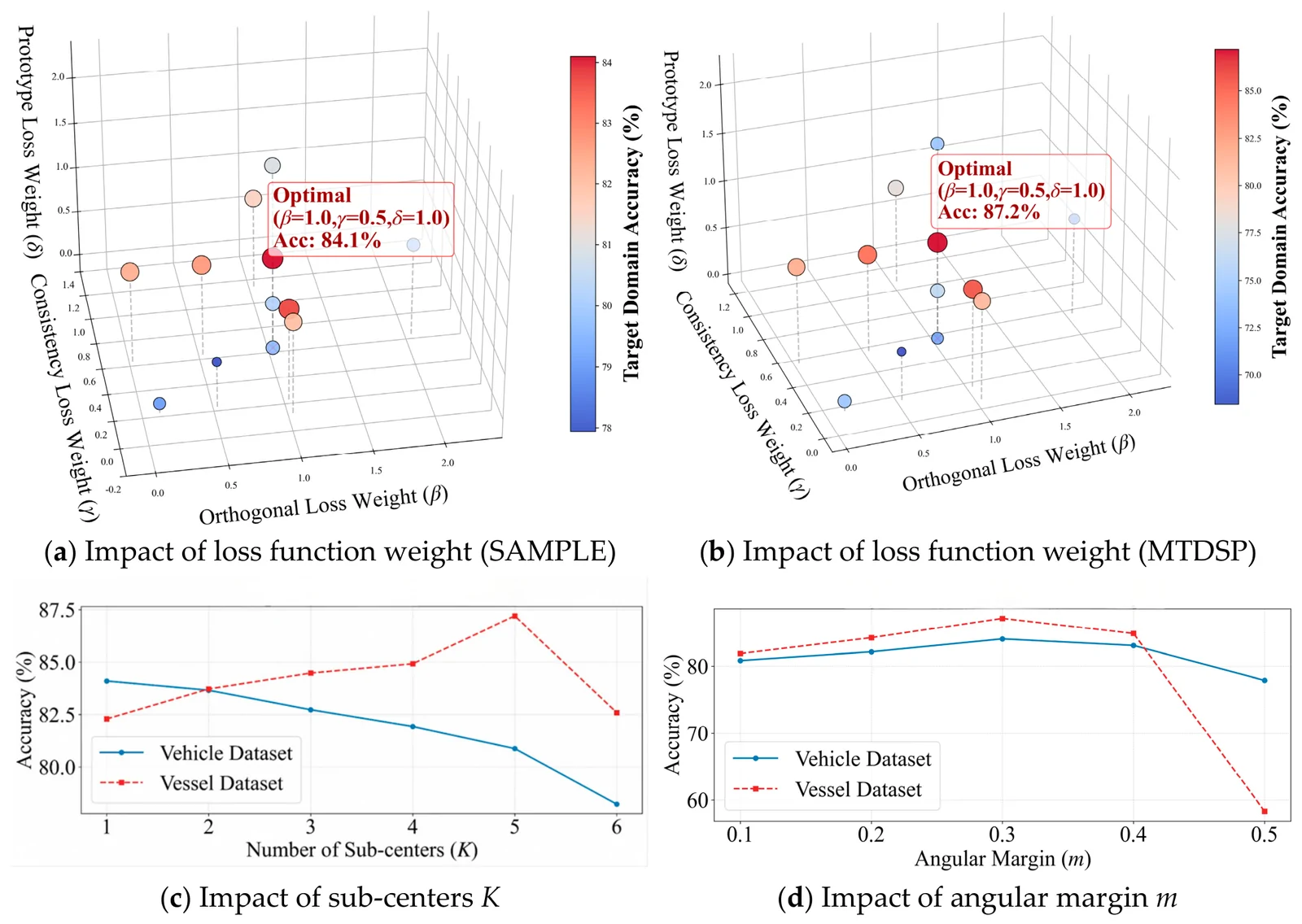
Parameter sensitivity comparison curves of the CROF-Net algorithm on SAMPLE/MTDSP datasets.

As illustrated in Table 14 and Figure 12a,b, the loss weights exert distinct effects on the two HRRP datasets. The ship dataset is more sensitive to weight fluctuations, and all loss items effectively promote model performance. The optimal weight combination is obtained to adapt to both vehicle and ship recognition tasks. Excessively large loss weights will over-regularize features and erase discriminative scattering information, while too small weights fail to suppress simulation-measurement domain offset, which explains the obvious accuracy drop of groups 3, 4, 6 and 7 in Table 14.

According to Figure 12c,d, the optimal sub-center number *K* varies with target type and scattering properties. Vehicle targets have compact single-group scattering structures with few strong scattering centers, so the feature distribution within each category presents an approximately single-cluster form; hence, the vehicle dataset achieves the best performance with *K* = 1. By contrast, ship targets are equipped with superstructures, masts, decks, and other multiple strong scattering components. When the radar observation azimuth changes, the position and amplitude of scattering peaks shift drastically, forming multiple separated feature manifolds within one category. Setting *K* = 5 allows independent sub-centers to fit scattering features under different azimuth sectors, which matches the inherent multi-modal scattering distribution characteristics of ships.

In addition, both datasets achieve the optimal performance at an angular margin of *m* = 0.3, demonstrating strong robustness of this parameter. A too small margin *m* cannot form sufficient isolation intervals between categories on the hypersphere and causes overlapping classification boundaries under clutter noise, whereas an overlarge margin excessively compresses intra-class feature space and reduces tolerance for attitude-induced scattering variations. The value *m* = 0.3 strikes a balance between inter-class separation and intra-class distribution tolerance for HRRP cross-domain recognition.

Experimental results indicate that vehicle targets are more suitable for single-center modeling with strong regularization, whereas ship targets require multi-sub-center modeling with weak regularization. By flexibly adjusting hyperparameters, the proposed CROF-Net can adapt to targets with different scattering characteristics, which verifies its good generalization ability and interpretability.

## 5. Conclusions

To address the simulation-measurement domain shift, scarce measured annotations, and complex electromagnetic clutter in HRRP recognition, this paper proposes a topological manifold-aligned cross-domain few-shot network (CROF-Net). The framework combines global distribution alignment and fine-grained prototype calibration to mitigate cross-domain distribution discrepancy. Built upon a SPA-IN-enhanced 1D-Conformer backbone, the network eliminates amplitude interference and extracts discriminative local scattering details and global topological features. Furthermore, an orthogonal regularized multi-sub-center ArcFace metric module is adopted to fit the attitude-induced intra-class multimodal distribution, effectively alleviating negative transfer and feature degradation in few-shot cross-domain training. Extensive experiments on SAMPLE and MTDSP datasets verify that CROF-Net outperforms conventional convolution networks, meta-learning methods, and state-of-the-art domain adaptation approaches. It achieves near-upper-limit recognition accuracy under small-sample and strong-clutter cross-domain conditions, exhibiting superior generalization, anti-interference robustness, and promising engineering application potential.

## Figures and Tables

**Table 1:** SPA-IN layer-wise dimensions of the 1D-Conformer network.

Layer	Input Dimension	Output Dimension
Primary 1D Conv + IN + GELU	(*B*, 1, 128)	(*B*, 64, 64)
1st SPA-IN Residual Block	(*B*, 64, 64)	(*B*, 128, 32)
2nd SPA-IN Residual Block	(*B*, 128, 32)	(*B*, 256, 16)
Dimension Permutation	(*B*, 256, 16)	(*B*, 16, 256)
Transformer Encoder	(*B*, 16, 256)	(*B*, 16, 256)
Dimension Restoration	(*B*, 16, 256)	(*B*, 256, 16)
Adaptive Global Average Pooling	(*B*, 256, 16)	(*B*, 256)

Note: Dimension format definition: (*B*, *C*, and *L*) denotes (Batch Size, Channels, and Sequence Length). All convolutional blocks adopt IN and GELU activation to mitigate domain shifts. No learnable positional encoding is artificially appended before the Transformer encoder, and SE channel attention is embedded in each residual block for adaptive scattering feature screening.

**Table 2:** The category and volume of the SAMPLE dataset (azimuth angle 10°~80°, elevation angle 14°~17°).

Category	Volume	Category	Volume	Category	Volume	Category	Volume	Category	Volume
2S1	174	M35	129	BTR70	92	M1	129	ZSU23	174
BMP2	107	M548	128	M60	176	T72	108	M2	128

**Table 3:** AIS auxiliary information display (select information at a specific instant of the echo acquisition).

AIS Information Item	AIS Information Value	Meteorological Information Item	Meteorological Information Value
AIS MMSI	200000000	Significant Wave Height (m)	0.2
Vessel Type	fishing boat	Wave Direction (°)	86
Nationality	China	Sea State Grade	2
Longitude (°)	37.7754	Wind Speed (m/s)	2.1
Latitude (°)	121.5064	Wind Direction (°)	256
Vessel Length (m)	15	Wind Force Grade	2
Vessel Beam (m)	3.1	Temperature (°)	27.3

**Table 4:** Parameters of X-band experimental VV polarization radars.

Radar System	Parameter	Radar System	Parameter
Operating Frequency Range	9.3~9.5 GHz	Transmit Peak Power	100 W
Detection Range	1/16~96 n mile	Antenna Rotation Speed (r/min)	2, 6, 12, 24, 48
Sweep Bandwidth	25 MHz (T2, T3)	Antenna Vertical Beamwidth	23°
Range Resolution	6 m	Antenna Length	2.5 m
Pulse Repetition Frequency (kHz)	1.6, 2.0, 3.0, 5.0, 10.0	Antenna Horizontal Beamwidth	1.1°
Transmit Waveform & Pulse Width	T1: Single Frequency 0.15 µs T2: Linear Frequency Modulation (LFM) 8 µs T3: Linear Frequency Modulation (LFM) 25 µs	Antenna Operating Mode	Circular Scan, Sector Scan, Fixed Pointing

**Table 5:** Number of samples for ship single polarization (There are 726 simulated data samples for each category).

Category	Measured Samples	Category	Measured Samples	Category	Measured Samples
200000000	7768	413203610	7288	412331720	7287
413021330	7205	413020540	7455	414150000	7377

**Table 6:** Recognition performance with different training strategies and few-shot sizes under the purely measured scenario (test set consists of measured data).

Training Data	Shot	Accuracy (%)	$D_{\mathrm{shift}\;}$ (↓)	$S_{\mathrm{intra}}$ (↑)
Simulation	0 (Lower Bound))	30.48	2.55	0.28
Measured	All (Upper Bound)	90.59	0.23	0.87
Measured	1	55.96	1.84	0.31
Sim/Measured	1	62.32	1.42	0.53
Measured	5	61.66	1.56	0.48
Sim/Measured	5	72.82	1.05	0.62
Measured	10	72.57	1.19	0.57
Sim/Measured	10	84.10	0.65	0.71

Note: In the tables, the symbol ↓ indicates that a smaller value of the corresponding metric means better algorithm performance, while ↑ indicates that a larger value represents better performance. This rule applies to all algorithms listed below.

**Table 7:** Recognition performance with different training strategies and few-shot sizes under the occlusion scenario (test set consists of occluded HRRP samples).

Training Data	Shot	Accuracy (%)	$D_{\mathrm{shift}\;}$ (↓)	$S_{\mathrm{intra}}$ (↑)
Simulation	All/0/0 (Lower Bound)	17.12	4.25	0.15
Measured	0/All/0	66.50	1.61	0.57
Occluded	0/0/All (Upper Bound)	95.53	0.12	0.80
Measured	0/1/0	31.44	3.19	0.24
Occluded	0/0/1	37.47	3.08	0.30
Sim/Measured	All/1/0	42.93	2.82	0.36
Sim/Occluded	All/0/1	53.35	2.29	0.44
Measured	0/5/0	45.91	2.74	0.38
Occluded	0/0/5	48.88	2.46	0.40
Sim/Measured	All/5/0	62.13	1.89	0.54
Sim/Occluded	All/0/5	71.71	1.34	0.62
Measured	0/10/0	64.61	1.86	0.53
Occluded	0/0/10	70.12	1.43	0.58
Sim/Measured	All/10/0	77.85	1.18	0.69
Sim/Occluded	All/0/10	81.94	0.79	0.71

**Table 8:** Recognition performance with various training strategies and few-shot sizes under noisy condition (test set consists of noisy HRRP samples).

Training Data	Shot	Accuracy (%)	$D_{\mathrm{shift}\;}$ (↓)	$S_{\mathrm{intra}}$ (↑)
Simulation	All/0/0 (Lower Bound)	13.41	4.92	0.14
Measured	0/All/0	62.28	2.02	0.57
Noisy	0/0/All (Upper Bound)	86.99	0.57	0.78
Measured	0/1/0	21.34	4.37	0.21
Noisy	0/0/1	23.57	4.30	0.23
Sim/Measured	All/1/0	34.22	3.78	0.33
Sim/Noisy	All/0/1	42.21	3.21	0.39
Measured	0/5/0	41.94	3.28	0.37
Noisy	0/0/5	52.07	2.73	0.48
Sim/Measured	All/5/0	56.38	2.34	0.51
Sim/Noisy	All/0/5	67.64	1.91	0.61
Measured	0/10/0	59.34	2.18	0.56
Noisy	0/0/10	70.96	1.56	0.62
Sim/Measured	All/10/0	72.51	1.42	0.64
Sim/Noisy	All/0/10	78.64	0.95	0.73

**Table 9:** Recognition performance with different training strategies and few-shot sizes under the purely measured scenario (test set consists of measured data).

Training Data	Shot	Accuracy (%)	$D_{\mathrm{shift}\;}$ (↓)	$S_{\mathrm{intra}}$ (↑)
Simulation	0 (Lower Bound)	16.77	12.22	0.01
Measured	All (Upper Bound)	90.59	0.18	1.02
Measured	1	23.65	10.08	0.10
Sim/Measured	1	63.91	4.52	0.61
Measured	5	51.86	6.19	0.49
Sim/Measured	5	83.36	0.96	0.86
Measured	10	72.83	2.58	0.78
Sim/Measured	10	87.20	0.23	0.93

**Table 12:** Comparison of recognition accuracy of various algorithms under different training strategies on the SAMPLE/MTDSP datasets (The bold values represent the optimal results).

Training data	Algorithm	SAMPLE Dataset Accuracy (%)			MTDSP Dataset Accuracy (%)		
		1-Shot	5-Shot	10-Shot	1-Shot	5-Shot	10-Shot
Measured Only	SVM [49]	22.45	41.22	56.35	18.57	28.31	45.18
	KNN [50]	18.31	35.66	48.91	15.16	25.42	38.64
	Template [51]	26.15	38.57	45.12	16.81	29.58	36.21
	1D-CNN [52]	15.24	32.45	53.45	12.35	28.63	48.42
	ResNet-1D [11]	13.84	36.32	58.15	11.61	31.21	54.51
	ViT-1D [53]	10.27	22.12	38.83	9.58	18.87	35.27
	Prototypical Networks [35]	38.45	50.57	52.24	28.47	45.55	56.38
	MAML [54]	35.11	49.85	60.55	25.85	43.62	52.17
	DANN [15]	14.49	35.15	59.28	12.61	29.24	55.82
	CROF-Net	**55.96**	**61.66**	**72.57**	**23.65**	**51.86**	**72.83**
Sim/Measured	SVM [49]	16.20	33.20	48.75	12.41	23.82	38.25
	KNN [50]	14.60	29.11	41.25	11.58	20.61	33.52
	Template [51]	18.25	30.82	39.30	14.65	25.45	31.18
	1D-CNN [52]	25.64	49.21	54.81	22.41	46.83	55.35
	ResNet-1D [11]	28.35	52.57	64.44	25.23	52.55	61.83
	ViT-1D [53]	21.26	45.92	62.55	18.58	39.41	59.55
	Prototypical Networks [35]	41.34	56.87	68.45	35.81	48.38	66.52
	MAML [54]	39.51	60.46	66.95	33.62	55.27	64.84
	DANN [15]	48.43	63.25	70.51	41.54	58.44	69.27
	CROF-Net	**62.32**	**72.82**	**84.10**	**63.91**	**83.36**	**87.20**

**Table 13:** Recognition accuracy of layered ablation experiments (MTDSP dataset), √ means the corresponding module is adopted, and × means the module is not adopted.

Exp. Name	EDA-Aug	SPA-IN	OMS-ArcFace	RAPC	1-Shot (%)	5-Shot (%)	10-Shot (%)
Base Line	×	×	×	×	22.41	46.83	55.37
G1	√	×	×	×	48.80	51.96	63.32
G2	√	√	×	×	53.93	68.05	76.17
G3	√	√	√	×	61.58	74.94	81.09
CROF-Net	√	√	√	√	63.91	83.36	87.20

**Table 14:** Results of the sensitivity analysis experiment regarding the weight parameters of loss functions.

Exp. Name	β	γ	δ	SAMPLE Dataset Accuracy (%)	MTDSP Dataset Accuracy (%)
1	0.5	0.5	1.0	82.63	84.54
2	1.0	0.1	1.0	83.71	85.48
3	1.0	0.5	0.5	80.25	76.16
4	2.0	0.5	1.0	79.46	71.23
5	1.0	1.0	1.0	81.52	78.21
6	1.0	0.5	2.0	80.88	74.39
7	0.5	0.1	0.5	77.94	68.45
8	0.1	0.1	0.1	79.06	74.50
9	0	0.5	1.0	82.28	81.77
10	1.0	0	1.0	81.95	81.07
11	1.0	0.5	0	79.72	72.44
CROF-Net	1.0	0.5	1.0	84.10	87.20

## Data Availability

The original data presented in the study are openly available in MTDSP at https://radars.ac.cn/web/data/getData?dataType=DatasetofRadarDetectingSea (accessed on 5 July 2026). and in SAMPLE at https://github.com/benjaminlewis-afrl/SAMPLE_dataset_public (accessed on 5 July 2026).
